# Institutional experience of in-stent stenosis after pipeline flow diverter implantation: A retrospective analysis of 6 isolated cases out of 118 patients

**DOI:** 10.1097/MD.0000000000025149

**Published:** 2021-03-19

**Authors:** Ting Wang, Seidu A. Richard, He Jiao, Junrao Li, Sen Lin, Changwei Zhang, Chaohua Wang, Xiaodong Xie, Chao You

**Affiliations:** a Department of Neurosurgery, West China Hospital, Sichuan University, 37 Guo Xue Xiang Street, Chengdu, PR China; b Department of Medicine, Princefield University, P. O. Box MA 128, Ho-Volta Region, Ghana West Africa; c Department of Radiology, West China Hospital, Sichuan University, 37 Guo Xue Xiang Street, Chengdu, PR China.

**Keywords:** aneurysm, intracranial, in-stent stenosis, malapposition, occlusion, pipeline embolization devices

## Abstract

Pipeline embolization devices (PLEDs) are flow diverting stents that have exhibited be safe and efficient in the treatment of complex aneurysms. Nevertheless, in-stent stenosis (ISS) has been reported as one of the cardinal complications associated with PLED. The association of wall malapposition and ISS in patient treated with PLED has not been reported.

A retrospective study was conducted to identify patients with ISS after implantation of PLED as treatment for intracranial aneurysms from April 25, 2018 to April 24, 2019. Incidence of ISS and its associated causes such as sharp change of the PLED, distal wall malapposition, inconsistent compliance between parent artery as well as the PLED occlusion due to intimal hyperplasia and vessel tortuosity. Assessment of conservative treatment and retreatment outcomes of ISS were documented.

In all, 6 ISS cases were identified by 2 independent neurointerventionalists out of 118 aneurysm patients treated with PLED. Thus, the incidence rate of ISS in patients treated with PLED was as low as 5% at our institution compared to other studies. The follow-up time for detection of ISS ranged from 6 to 12 months after implantation. Several combinations of reasons such as sharp change of the PLED, distal wall malapposition, inconsistent compliance between parent arteries as well as PLED occlusion due to intimal hyperplasia and vessel tortuosity accounted for the causes of ISS during our analysis. Conservative treatment with a combination of antiplatelet during follow-ups did not resolve the ISS in our study probably due to associated underlying factors above.

## 1 Introduction

Pipeline embolization devices (PLEDs) are flow diverting stents that have exhibited be safe and efficient in the treatment of complex aneurysms.^[1–3]^ They are capable of altering the hemodynamics of the parent vessel as well as the aneurysmal sac by diverting blood inflow from the sac and triggering endothelialization across the neck of aneurysm.^[4–6]^ Nevertheless, associated morbidity and mortality rates are about 2.8% to 14.1% and 0% to 3.7%, respectively, with a permanent morbidity rate of 1.3% to 6.3%.^[3,7,8]^

PLED have showed to be highly effective in absolute delay occlusion of certain brain aneurysms; nevertheless, complications associated with their implantations are numerous.^[1,3,6,9]^ These includes, side branch occlusion, delayed aneurysmal rupture, as well as delayed intraparenchymal hemorrhage.^[4,5,10]^ Also, in-stent stenosis (ISS) has been reported as one of the cardinal complications associated with PLED.^[5,11,12]^ Nevertheless, no data exist on the association of wall malapposition and ISS in patient treated with PLED.

Our retrospective analysis report finding of ISS and its associated causes such as sharp change of the PLED, distal wall malapposition, inconsistent compliance between parent artery as well as the PLED occlusion due to intimal hyperplasia and vessel tortuosity. We also assessed the retreatment as well as no retreatment outcomes of ISS.

## 2 Methods

### 2.1 Patients

A retrospective study was conducted to identify patients with ISS after implantations of Pipeline Flex embolization devices (PLED; Medtronic, Neurovascular, Irvine, CA) as treatments for intracranial aneurysms from April 25, 2018 to April 24, 2019. We analyzed their demographic data, aneurysm sizes and locations, the sizes of the PLEDs used with or without coils. The detection, characteristic, classification, treatment as well as follow-up evaluation of ISS were documented. Also, associated malapposition and aneurysmal occlusion in patients with ISS were also documented. The research ethical committee of our hospital approved this study. On follow-up visits, the patients as well as their relatives were dually informed about our intention to involve them in a study and they fully concerted to the use of their information. Written informed consents were obtained from all the patients and their relatives.

### 2.2 Grading of in-stent stenosis

ISS was defined as any lumen loss within the implanted PLED which appears like a gap between the opacified vessel lumen and the inner contour of the metallic mesh angiographically. ISS was considered absent if there was no such gap on angiography. ISS was graded from mild (25%–50%), moderate (50%–75%) to severe (>75%). If the gap was <25% of the vessel lumen, intimal hyperplasia was established.^[1]^ The PLEDs were also divided in proximal, middle as well as distal according anatomical location. This anatomical division was used to assess the location of associated wall malapposition in the patients with ISS. Bouthillier's classification of internal carotid artery segments was used to determine the location of the aneurysms.^[13]^

### 2.3 Antiplatelet regimen

All patients were given 100 mg of aspirin and 75 mg of clopidogrel daily for at least 5 days prior to the procedures. P2Y12 assay (Verify Now, Accumetrics, San Diego, CA) were utilized to check antiplatelet inhibitory efficiency. If the inhibition effect was insufficient, clopidogrel was replaced with 90 mg of ticagrelor twice daily. Dual-antiplatelet therapy was continued for 6-months after the operation and aspirin alone continued for 12-months based on our protocol.

### 2.4 Endovascular treatment

The entire endovascular procedures were carried out under general anesthesia. Patients informed consents were obtained before the procedures in all cases. Digital subtraction angiographies (DSAs) were performed prior to the endovascular procedures, and the locations as well as sizes of the aneurysms in the internal carotid arteries (ICAs), middle cerebral arteries (MCAs), and vertebral arteries (VAs) identified and quantified. In all patients, after securing a line in the femoral artery, a triaxial system consists of 9F short femoral sheath, 7F 90 cm long sheath and 5F Navien intermediate catheter were implanted in the petrous/cavernous segments of the ICAs or extracranial segments of the VAs. Normal saline was continuously infused to prevent any ischemic events after femoral sheaths were placed successfully in all the patients. The 7F long sheaths were advanced into the extracranial segments of the ICAs or VAs to obtain frontal views and lateral views via 3-dimension angiographies. The PLEDs sizes were determined by measuring the diameters and lengths of parent arteries with FD-20 software (Philips Healthcare, Eindhoven, The Netherlands). The PLEDs were deployed via Marksman microcatheters (Medtronic Neurovascular, Irvine, CA).

### 2.5 Intra-operation and follow-up angiographic evaluation

Contrast retentions in the aneurysms, wall appositions, compliance between stents and parent arteries, patency and blood flow restrictions of parent arteries were evaluated immediately after PLED implantations and during follow-ups. Angiographic follow-ups with dilute contrast enhanced high-resolution Xper-CT (Philips Healthcare, Eindhoven, The Netherlands) were scheduled at 3, 6, 9, and 12 months after PLED placements. Aneurysmal occlusions were classified according to the Raymond-Roy occlusion classification (ROC) into 3 groups: complete occlusion (ROC1), neck remnant (ROC2), and residual aneurysm (ROC3).

### 2.6 Inclusion and exclusion criteria

Patients with ISS after treatment with PLED were strictly included in this study. Also, only patients with at least 6-months DSA follow-up were eligible for the study. Nevertheless, patients in whom gap inside the implanted PLED <25 were excluded.

## 3 Results

In all, 6 cases (Table 1) were defined as ISS by 2 independent neurointerventionalists with consistency out of 118 aneurysm patients treated with PLEDs in the stipulated time period. Therefore, we observed 5% (6/118) incidence rate of ISS in intracranial aneurysmal patients treated with PLEDs at our institution. The patients were made up of 2 males and 4 females with a mean age of 42. Only one of the patients was a child while the rest where adults. Three of the patients were asymptomatic according to our analysis while 2 presented with dizziness and 1 with blurring of vision. Based on Bouthillier's classification of ICA segments, aneurysms where located in C4 segment in 2 patients and C6 in 3 patients. Only 1 patient had an aneurysm in the B-V4 segment of the vertebral artery. The largest aneurysm measured 30.8 × 26 mm while the smallest measured 2.5 × 1.3 mm. One patient had vertebral dissecting aneurysm (Table 1).

**Table 1:** Patient demographics, aneurysmal characteristics and follow-up results.

Case	Clinical Presentation	Coils	Aneurysm size	Aneurysm location	PLED size	Malapposition	Location of malapposition	Follow-ups (month)	Aneurysm occlusion	ISS	Aggravation of ISS	Retreatment	Resolved
1	Incidence	N	6.3∗7.2	L-C4	4.0∗30	N	N	12	ROC1	severe	Y	N	N
2^∗^	Dizziness	N	30.8∗26	R-C4	3.75∗35 (dropped) 4.0∗35 / 4.75∗35 /5.0∗30	N	N	12	ROC1	moderate	Y	Y	Y
3	Incidence	N	3.6∗4.7 2.5∗1.3	R-C6	5.0∗25	N	N	6	ROC3	moderate	N	N	N
4	Incidence	N	8.48∗5.8	R-C6	5.0∗25	Y	distal	9	ROC3	mild	N	N	N
5	Blurred vision	Y	19.8∗19	L-C6	4.5∗20	N	N	6	ROC1	mild	N	N	N
6	Dizziness	N	DA	B-V4	4.75∗35	Y	distal	6	ROC3	moderate	N	N	N

DA = dissection aneurysm, ISS = in-stent stenosis, N = no, ROC = Raymond–Roy occlusion classification, Y = yes, Bouthilliers segment of internal carotid artery: C4 cavernous; C5 clinoid; C6 ophthalmic. Three PLEDs were implanted successfully in case 2∗, the pipeline of size 3.75∗35 dropped into the aneurysmal sac. A total of 6 case of ISS were identifies out of 118 patients treated with PLEDs. The incidence rate was 5%.

Endovascular treatments were successful in all patients without procedure-related complications. However, in case 2, the first implanted PLED dropped into the aneurysmal sacs so 2 more PLEDs with larger sizes were implanted again. Case 3 had bilateral aneurysms. PLED implantation with coiling was done in 1 patient while the remaining 5 patients received only PLEDs implantations. Associated wall malapposition was observed in 2 patients. In both patients, the wall malappositions occurred in the distal segments of the ICAs. Three patients had ROC1 aneurysmal occlusion while the remaining 3 had ROC3 (Table 1).

The follow-up time for detection of ISS ranged from 6 to 12-month after implantation. One case was rated as severe ISS on 6-month follow-up. The patient's degree of ISS deteriorated on 12-month follow-up visit without any ischemic events and no remedial measures were taken because of patients’ refusal of further treatment (Table 1). Moderate ISS was identified in 3 cases in the first follow-up DSAs, of which 1 case aggravated into severe ISS accompanied by obvious flow restriction on the second follow-up imaging. Balloon angioplasty and neuroform EZ 4.5∗20 were used to retreat this patient and the ISS resolved. The patient's ISS was dramatically improved on 12 months follow-up visits (Table 1). Two patients had mild ISS which did not aggravate on all follow-up visits and thus these patients were not retreated. Nevertheless, their ISS did not resolve too (Table 1).

**Figure 1:**
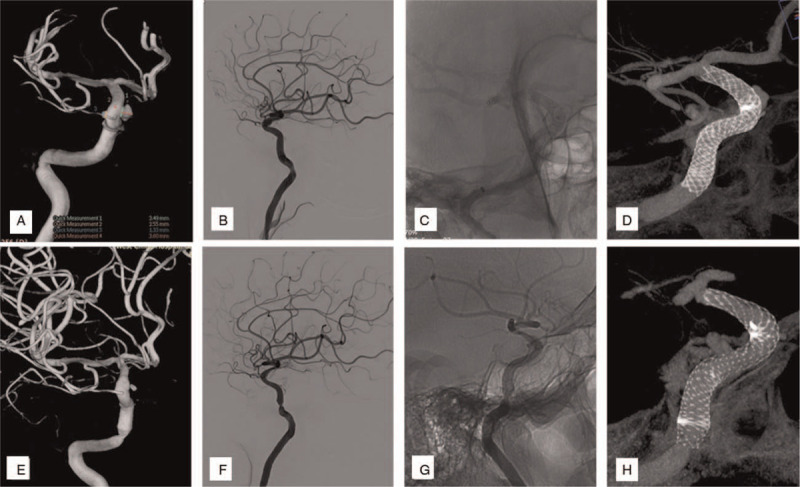
(Case 2): Are images associating shape change of PLED as the cause of ISS. A: shows 2 aneurysms in the right ophthalmic segment of internal carotid artery (ICA). B: shows the lateral view of the parent artery. C: Shows un-subtracted image of implanted PLED intraoperatively. D: is a diluted contrast Xper-CT image of the stent intraoperatively. E: shows a little residual in 1 aneurysm while another was completely occluded on 6-month follow-up images. F&G: Are lateral view of parent artery showing vessel lumen stenosis. H: shows the proximal shape change of the stent on a diluted contrast Xper-CT image.

**Figure 2:**
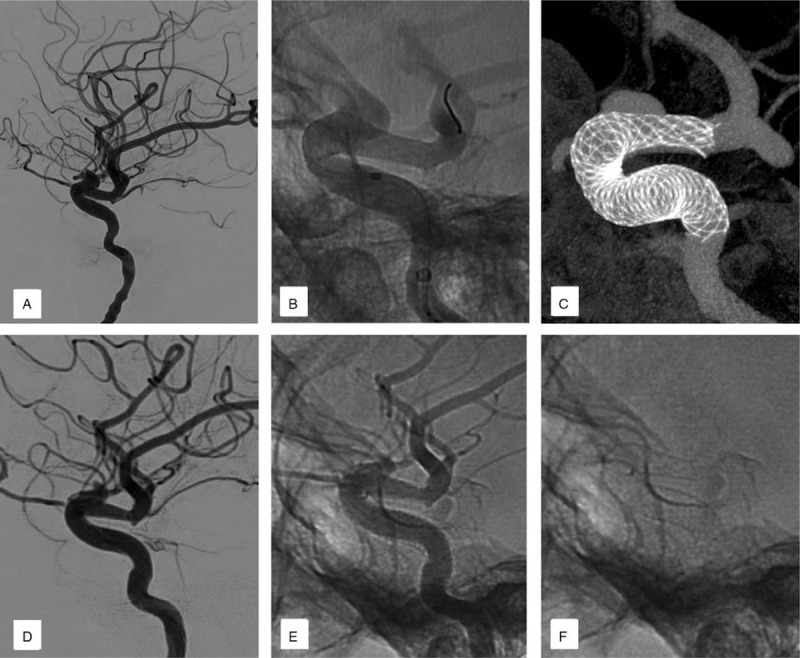
(Case 4): Are images demonstrating the association of distal malapposition of PLED as the cause of ISS. A: displays the ophthalmic segment aneurysm of right ICA intraoperatively. B: shows the distal end of PLED intraoperatively. C: Shows distal malapposition of the stent in diluted contrast Xper-ct image. Fig 2d-f were 6-month follow-up angiography of lateral views. D: shows loss of vessel lumen in the distal end of PLED on 6-month follow-up angiograph. E: Is an un-subtracted image showing the relationship between the opacified vessel lumen and the inner contour of the metallic mesh. F: shows the shape of the stent.

**Figure 3:**
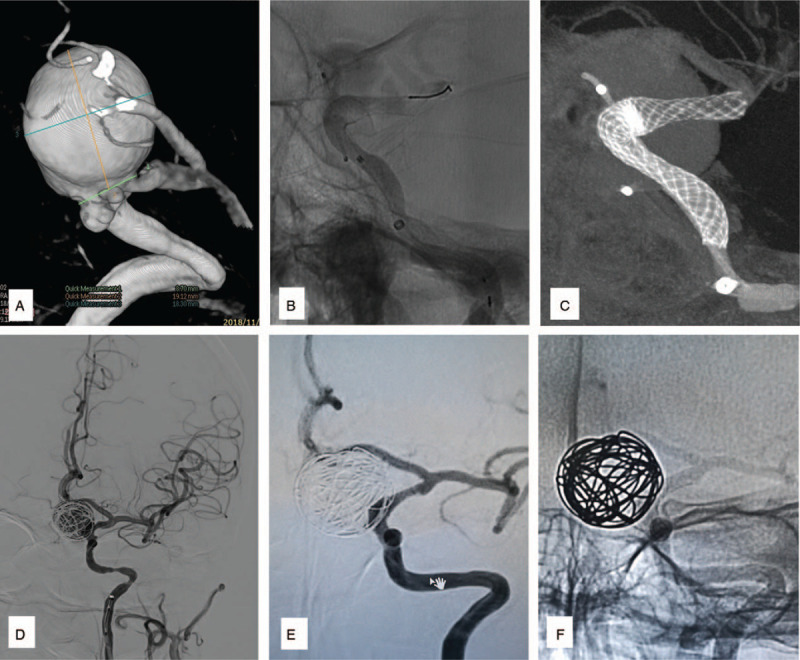
(Case 5): Are images demonstrating the association of inconsistent compliance between parent artery and the PLED as the cause of ISS. A: show the size and location of the aneurysm. B: show inconsistent compliance between parent artery and the distal end of the stent before coiling. C: is the diluted contrast Xper-ct image showing the inconsistent compliance clearer. D: show no stenosis in the distal end of the stent immediately after the procedure. E: display stenosis at the site of inconsistent compliance on 6-month follow-up images. F: is a casted image showing the inconsistent compliance on 6-month follow-up.

**Figure 4:**
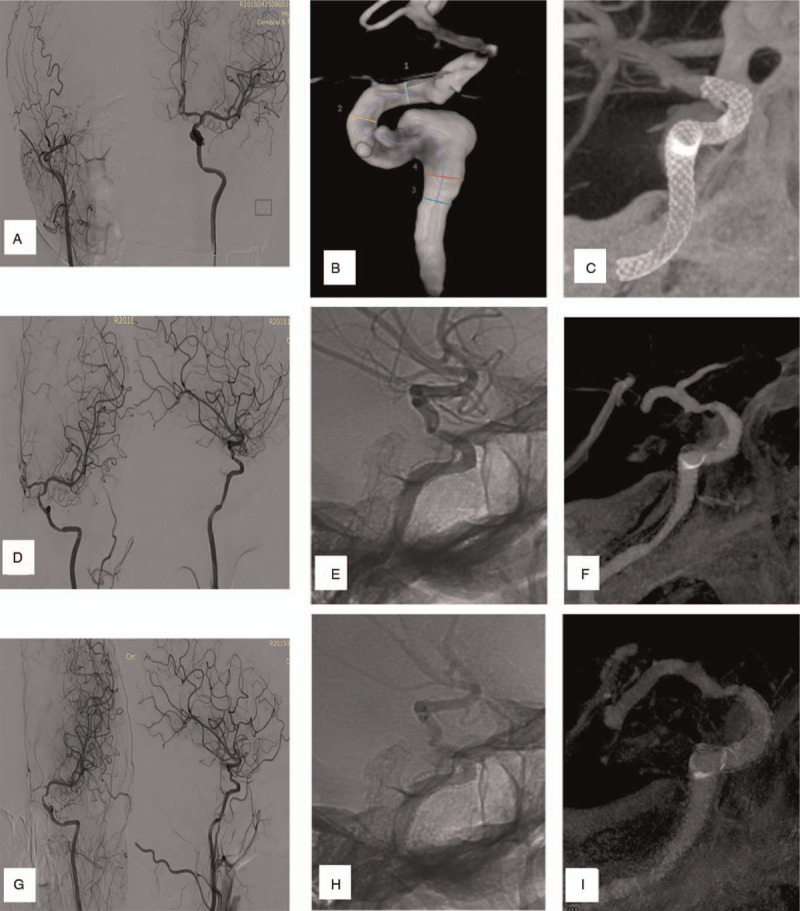
(Case 1): Are imaging showing the PLED occlusion due to intimal hyperplasia and vessel tortuosity as cause of ISS. A: shows the frontal views of bilateral ICA. Initially, the right was occluded while the left A1 segment of anterior cerebral artery was patent with compensation from the contralateral anterior communicating artery. B: shows the aneurysm in cavernous segment of ICA. C: shows the shape of the stent as well as confirmation that, the stent covered the aneurysm centrally. D: is 3-months follow-up frontal and lateral views of left ICA showing ISS at the distal and proximal end of the stent. The ISS is severe at the distal end while the aneurysm is completely occluded. E: Shows diffuse intimal hyperplasia inside the stent. F: shows tortuosity of terminal segment of ICA. G: Is a 12-month follow-up frontal and lateral views of left ICA showing obvious flow restriction of left anterior cerebral artery an obstructed anterior communicating artery. The ISS of proximal end was relieved to some extent. H&I: shows diffuse intimal hyperplasia is still present.

Several combinations of reasons were speculated as the cause of ISS during our analysis. Shape change was responsible for 2 cases (Case 2 & 3; Fig. 1, A-H), while distal malapposition of PLED was considered as the cause in 2 cases (Case 4 & 6; Fig. 2, A-F). Inconsistent compliance between parent arteries and stents were reasons for ISS in the remaining 2 cases (Case 1 & 5, Fig. 3, A-F). Furthermore, PLED stenosis due to intimal hyperplasia and vessel tortuosity was observed in Case 1 (Fig. 4, A-I).

## 4 Discussion

Flow redirection and tissue overgrowth are 2 main mechanisms via which PLED works.^[4]^ PLED is able to bridges the aneurysm neck and decrease the blood flow into the aneurysm sac, yet delivering blood to adjacent perforators as well as side branches. Flow stasis as well as formation of a stable aneurysmal thrombus are triggered by decrease in blood circulation within the aneurysm and accelerates necessary aneurysm occlusion.^[4,14]^ Nevertheless, side branch occlusion, delayed aneurysmal rupture, as well as delayed intraparenchymal hemorrhage are still observed as complications associated with PLED.^[10]^ Also, ISS has been reported as one of the cardinal complications associated with PLED.^[5,11,12]^

Although the incidence of ISS was been estimated by various research findings to be above 10% but less than 40%, we observed 5% incidence rate in patients treated with PLED at our institution. Cohen et al observed an incident rate of 38% in patients they treated with Silk Flow Diverter stents and 39% in patients they treated with PLEDs.^[5]^ Zhou et al found an ISS rate of 10.1% in their meta-analysis.^[3]^ Occurrence of ISS is therefore very minimal in patients treated with PLED in our institution. Furthermore, Cohen et al observed signs of ISS in 38% of their patients on initial 2-month angiographic follow-up of patients who were treated with PLED or Silk Flow Diverter stents.^[5]^ We detected ISS on follow-up in 3 of our patients on the six-month follow-up, while in the remaining 3 patients, 1 developed ISS on 9-month and 2 patients developed ISS on 12-months follow-ups. Nevertheless, none of patients develop ISS on the first 3 months follow-up visits which means that ISS often do not occur at early stages of PLED implantation but rather a later occurrence.

Three of the patients were asymptomatic according to our analysis while 2 presented with dizziness and 1 with blurring of vision. Cohen et al observed that, ISS was asymptomatic in 12 out of 13 of their patients.^[5]^ The best combination and exact duration of prophylactic platelet inhibition drugs has not been established, which has stressed the need for enhance confirmation for modifying antiplatelet therapy.^[3,15,16]^ Cases of severe ISS have proven to be partially reversible with conservative treatment such as short-term increase in the dose of antiplatelet therapy.^[5,8]^ Diverter angioplasty has also proven to resolve end tapering with minor ISS.^[5]^ All our patients were given 100 mg of aspirin and 75 mg of clopidogrel daily for at least 5 days prior to the procedures. Dual-antiplatelet therapy was continued for 6-months after the operation and aspirin alone continued for 12-months based on our protocol. Nevertheless, our study revealed that, ISS would usually not resolve with the above conservative treatment. Five of our patients with various degrees of ISS were treatment conservatively and their ISS did not resolve. However, 1 patient was retreated and the ISS resolve on follow-up angiography.

Lubicz et al assessed ISS by measuring the gap between the contrast filled vessel lumen and the inner silhouette of the stent,^[17]^ whilst Ruben et al assessed ISS by associating vessel lumen on follow-ups with the primary angiograms.^[18]^ In our study, ISS was defined as any lumen loss within the implanted PLED which appears like a gap between the opacified vessel lumen and the inner contour of the metallic mesh angiographically as demonstrated by Lubicz et al. ISS was considered absent if there was no such gap on angiography. Xiang et al indicated that, torpid aneurysmal flow and exceptionally low wall shear pressure may trigger aneurysmal wall degradation via unknown inflammatory pathways resulting into ISS.^[19]^ Cohen et al further indicated that, instant angiographic display of ISS as well as good prognosis of patients whose antiplatelet agents’ doses were increased indicate a characteristic biological behavior that is very different from the ISS that was originally associated with neointimal hyperplasia.^[5]^ In case 1, we observed diffuse intimal hyperplasia inside the stent as well as tortuosity of terminal segment of ICA.

Zhou et al indicated that the aneurysm is not instantaneously shielded after treatment with flow diverting devices which frequently result in total occlusion in about 3 to 12 months.^[3]^ Using the ROC, we observe total occlusion of the aneurysms sacs in 3 patients (ROC1) while the remaining 3 patients had residual aneurysm sacs (ROC3). We are thus unable to postulate the association of aneurysmal occlusion or non-occlusion on the development of ISS. Zhou et al further compared the PLED with coil embolization as well as stent-assisted coiling and observed that, PLED implantation did not demonstrate any substantial variance in morbidity as well as mortality between the 2 treatment modalities.^[8]^ In our analysis we only identified 1 patient treated with coil embolization who subsequently developed ISS while 5 of the patients treated with only PLED developed ISS. Although we did not get equally proportion of patients treated with both modalities, we think patients treated with PLED have higher chances of developing ISS. It is estimated that, about 2.5% of aneurysms treated with coils often results in complications such as coil prolapse into the parent vessel as well as coil penetration.^[20]^

The precise time for total occlusion of an aneurysm after PLED treatment is unspecified. Nevertheless, it is proven that, early aneurysm occlusions are essential in preventing complications. Also, overlapping of the devices may lead to possible occlusion of the side branches or perforating arteries often resulting in secondary ischemic hitches after the procedure.^[21]^ Lubicz et al recommended a stent diameter that is 0.25 to 0.5 mm larger than the distal parent vessel diameter.^[22]^ We experience technical challenges in case 2 because of the size of the implanted PLED. The PLED was relatively shorter than aneurysm so it dropped into the aneurysmal sac. With this experience, we support Lubicz et al view that the stent diameter should be about 0.25 to 0.5 mm larger than the distal parent vessel diameter.

Cohen et al observed 2 patients with severe segmental stenosis which they efficaciously managed with conservative measures.^[5]^ We observed severe stenosis in Case 1 in whom we managed conservatively with no improvement. Nevertheless, in Case 2, balloon angioplasty was used to correct the stenosis with a good outcome. Cohen et al observed that, only angioplasty balloons, but not low-pressure balloons, were capable of reopening tapered diverter end, which were entrenched in the arterial wall.^[5]^ Several studies have demonstrated that, malapposition can result in endoleaks as well as incomplete aneurysm occlusion.^[11,23,24]^ Also, late thrombosis as a result of poor apposition can augment the risk of secondary thromboembolic complications.^[11,14,25]^ Pérez et al delineate the potential role malapposition has on the incidence of ISS.^[11]^ Nevertheless, they did not indicate it occurrence in their study. We detected the occurrence of malapposition and ISS in 2 of our cases (4&6). In both cases, the malapposition was located distally. The ISS was however mild in case 4 but rather moderate in case 6.

Several combinations of reasons accounted for the causes of ISS during our analysis. Shape change was responsible for 2 cases, while distal malapposition of PLED was considered as the cause in 2 cases. Inconsistent compliance between parent arteries and stents were reasons for ISS in the remaining 2 cases. Furthermore, the susceptibility of vessel occlusion due to diffuse intimal hyperplasia inside the stent as well as tortuosity of terminal segment of ICA was observed in Case 1. Nevertheless, it has been demonstrated that, the underlying lower incidence of ISS after flow diversion are probably numerous, with stent composition, flow rates, wall shear stress, as well as underlying molecular differences between the cerebrovascular architecture being key factors.^[6]^ Intimal damage by the device often results in vascular smooth muscle cell (SMC) stimulation, leading to the opening of stretch-responsive Ca^2+^ channels.^[6]^ Also, stent oversizing was directly implicated in the magnitude of neointimal hyperplasia.^[6]^

Three different cellular reactive phases often occur following mechanical vascular injury instantly after stent placement. The early phase usually involves platelet activation as well as inflammation while the intermediate phase involves granulation of tissue, SMCs migration as well as proliferation. The late phase however involves tissue remodeling.^[11,26,27]^ It was proven that, paracrine signaling results in simultaneous endothelial cell activation, release of vasoactive as well as procoagulant substances during cell proliferation.^[28]^ It is also proven that; stenotic tissue composes of SMCs as well as extracellular matrix without any endothelial cells.^[29]^ Furthermore, endothelial cells have been associated with neointimal growth along flow diverter device.^[30,31]^

## 5 Conclusion

The incidence of ISS was relatively very low in our study involving higher numbers of patients treated with aneurysms as compared to earlier studies. Wall malapposition was associated with the occurrence of ISS. Conservative treatment with a combination of antiplatelet during follow-ups did not resolve the ISS in our study probably due to associated underlying factors such as sharp change of the PLED, distal wall malapposition, inconsistent compliance between parent arteries as well as PLED occlusion due to intimal hyperplasia and vessel tortuosity.

## Author contributions

**Conceptualization:** Ting Wang, Seidu A Richard, He Jiao, Junrao Li, Sen Lin, Changwei Zhang, Chaohua Wang, Xiaodong Xie, Chao You.

**Data curation:** Ting Wang, Seidu A Richard, He Jiao, Junrao Li, Sen Lin, Changwei Zhang, Chaohua Wang, Xiaodong Xie, Chao You.

**Formal analysis:** Ting Wang, Seidu A Richard, He Jiao, Junrao Li, Sen Lin, Changwei Zhang, Chaohua Wang, Xiaodong Xie, Chao You.

**Funding acquisition:** Changwei Zhang, Chaohua Wang.

**Methodology:** Ting Wang, Seidu A Richard, He Jiao, Junrao Li, Sen Lin, Changwei Zhang, Chaohua Wang, Xiaodong Xie, Chao You.

**Resources:** Chaohua Wang, Xiaodong Xie.

**Supervision:** Changwei Zhang.

**Writing – original draft:** Ting Wang, Seidu A Richard.

**Writing – review & editing:** Ting Wang, Seidu A Richard, He Jiao, Junrao Li, Sen Lin, Changwei Zhang, Chaohua Wang, Xiaodong Xie, Chao You.
